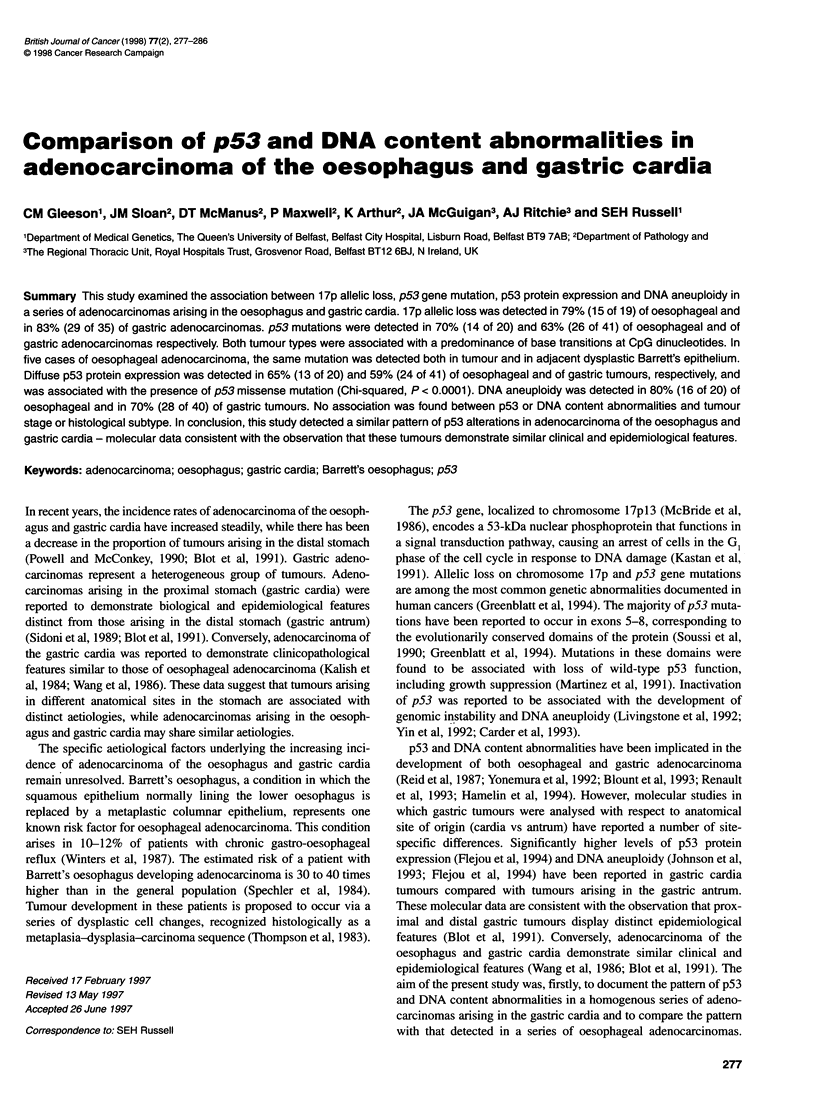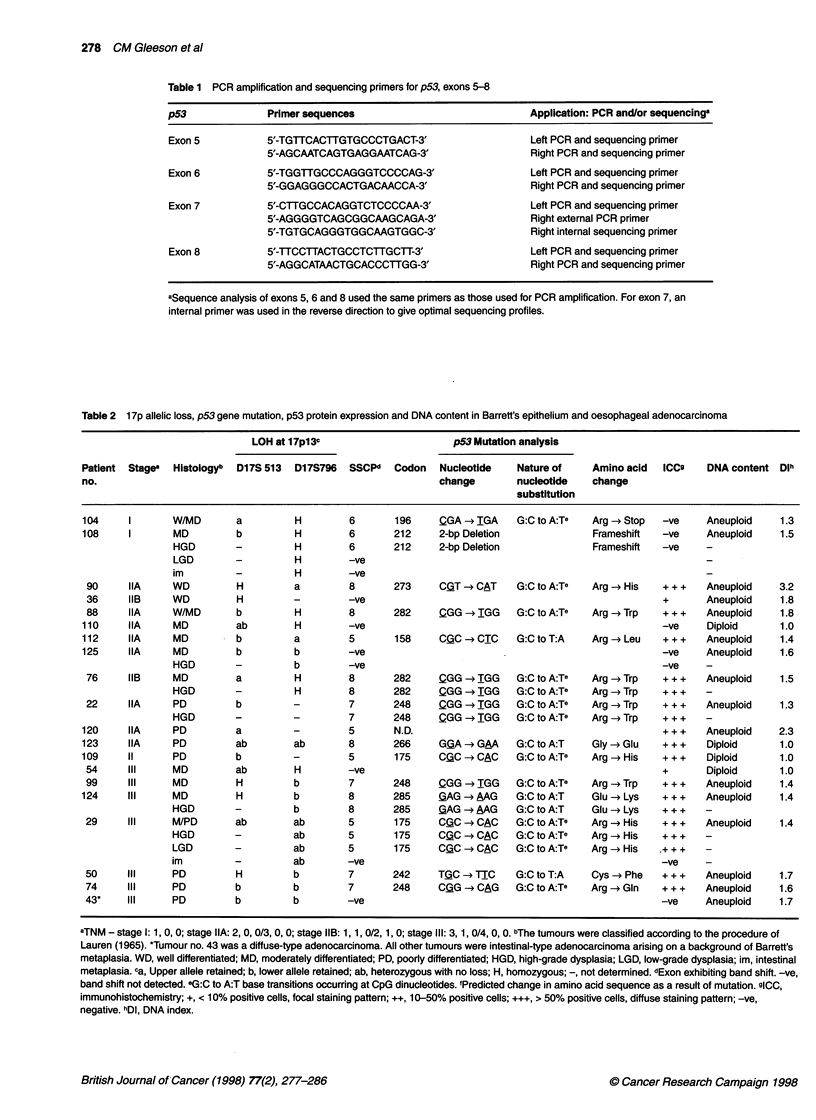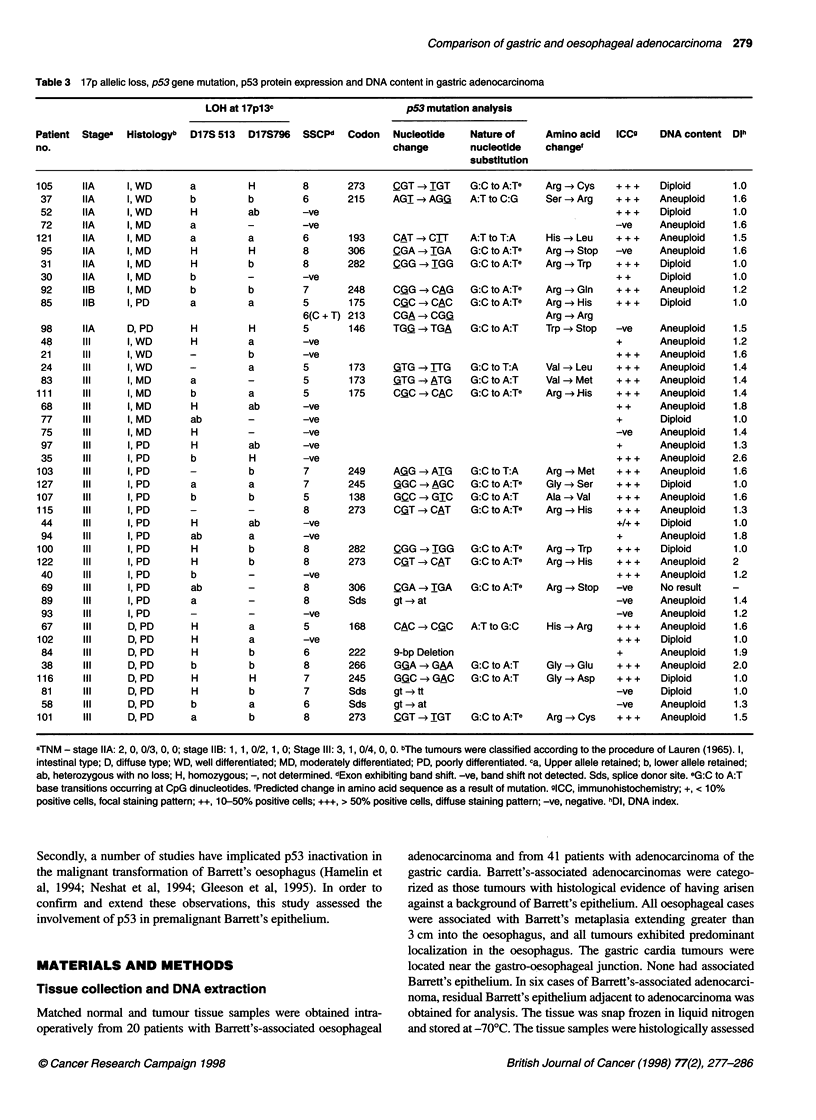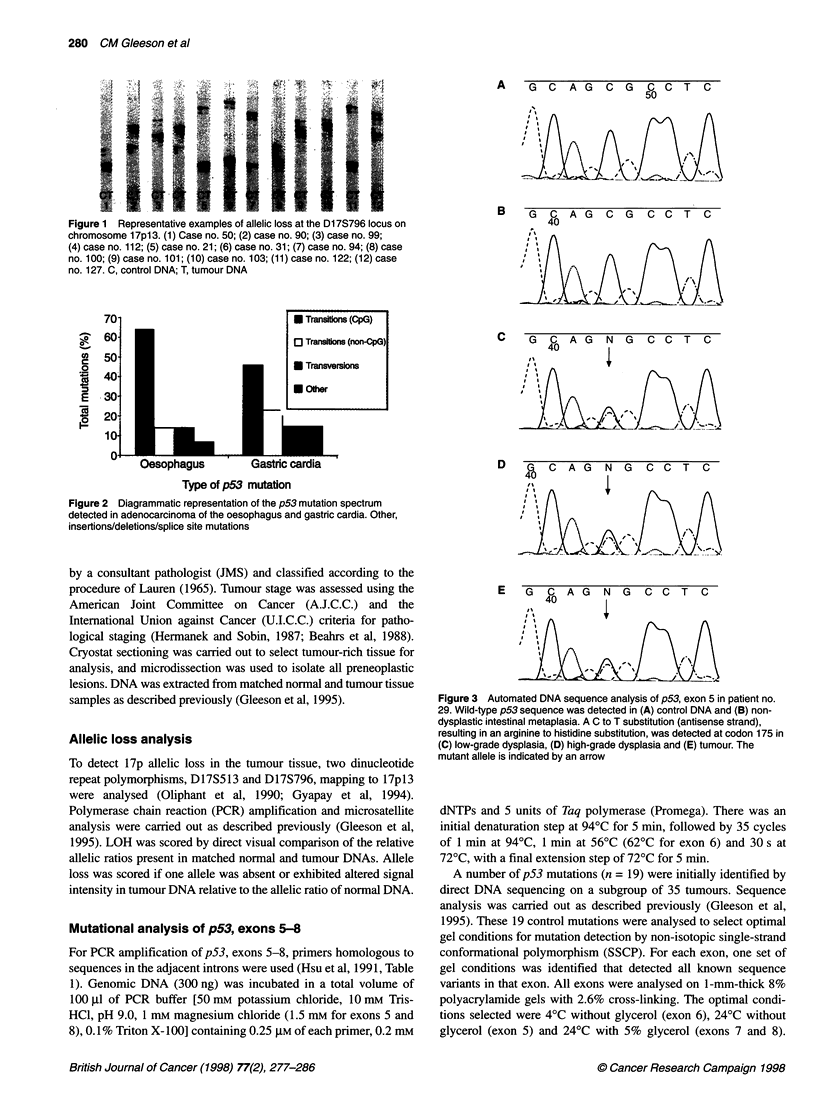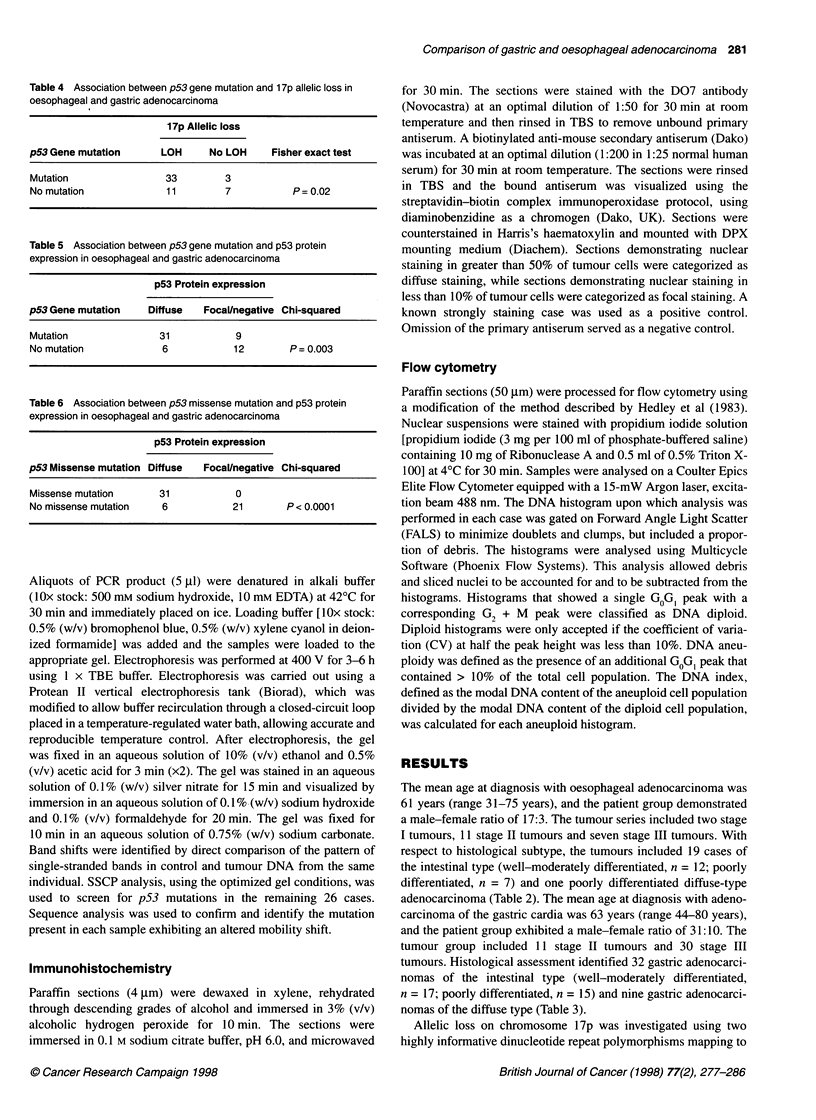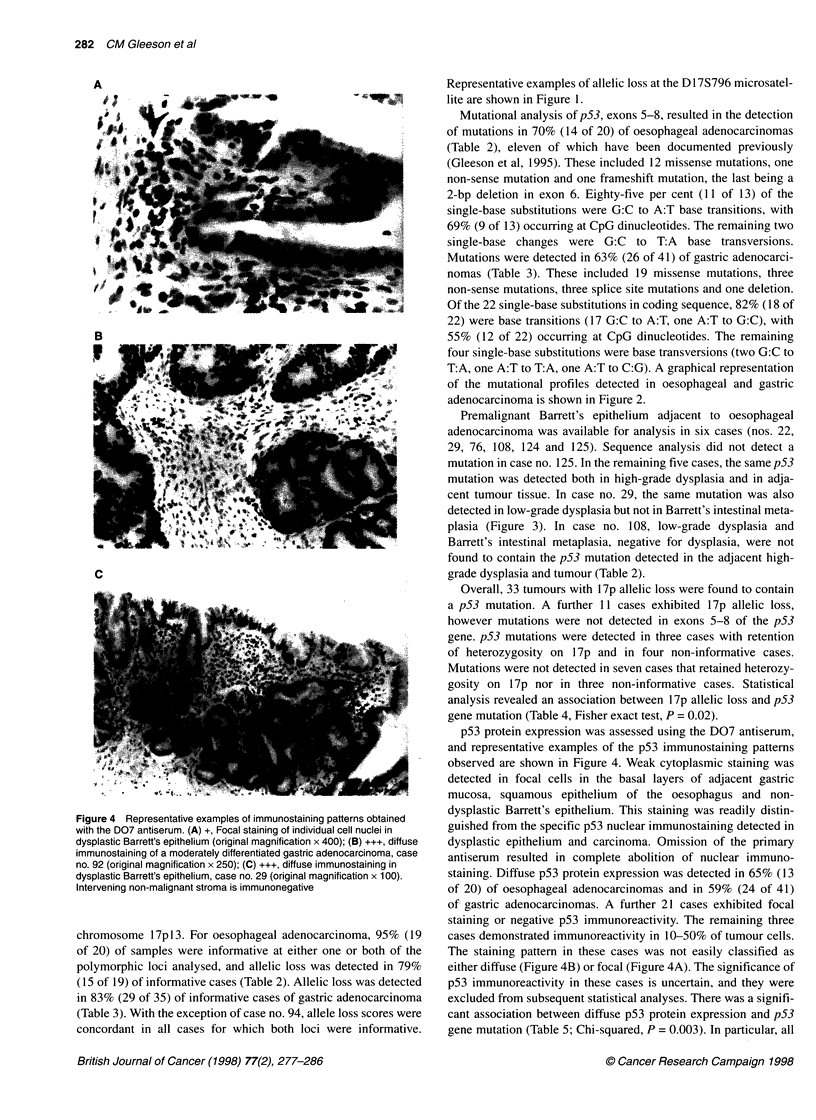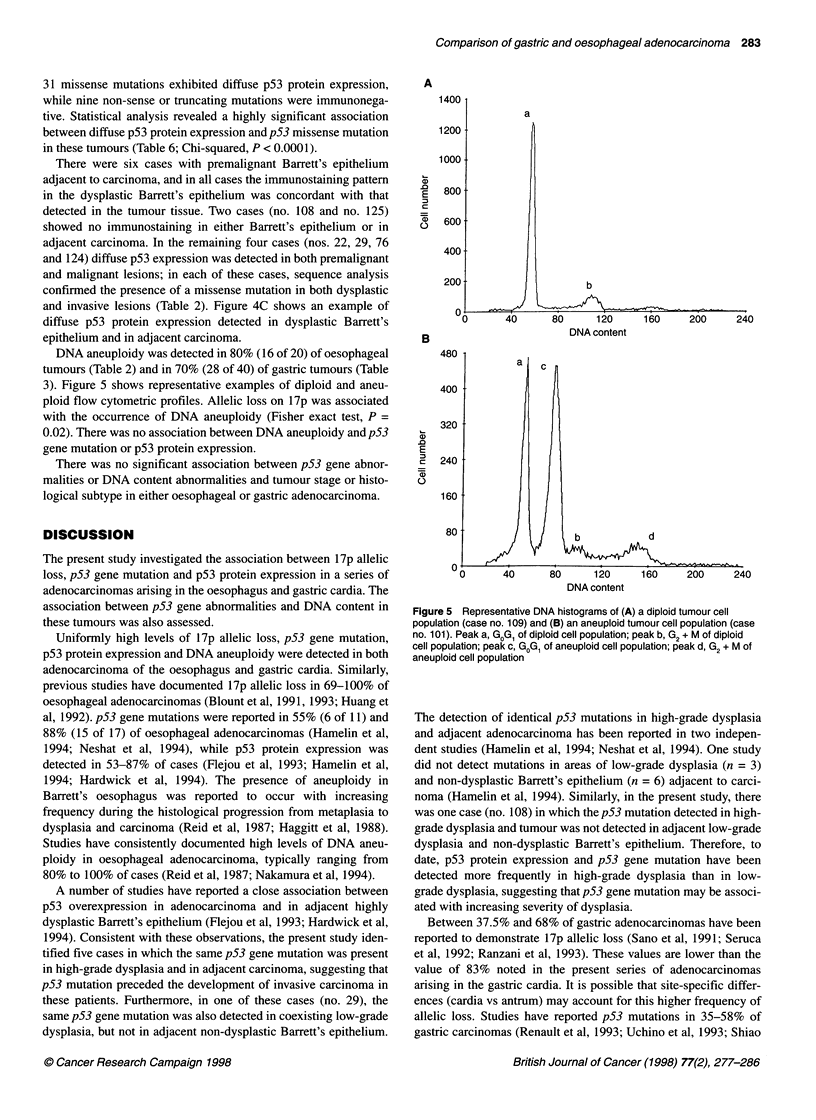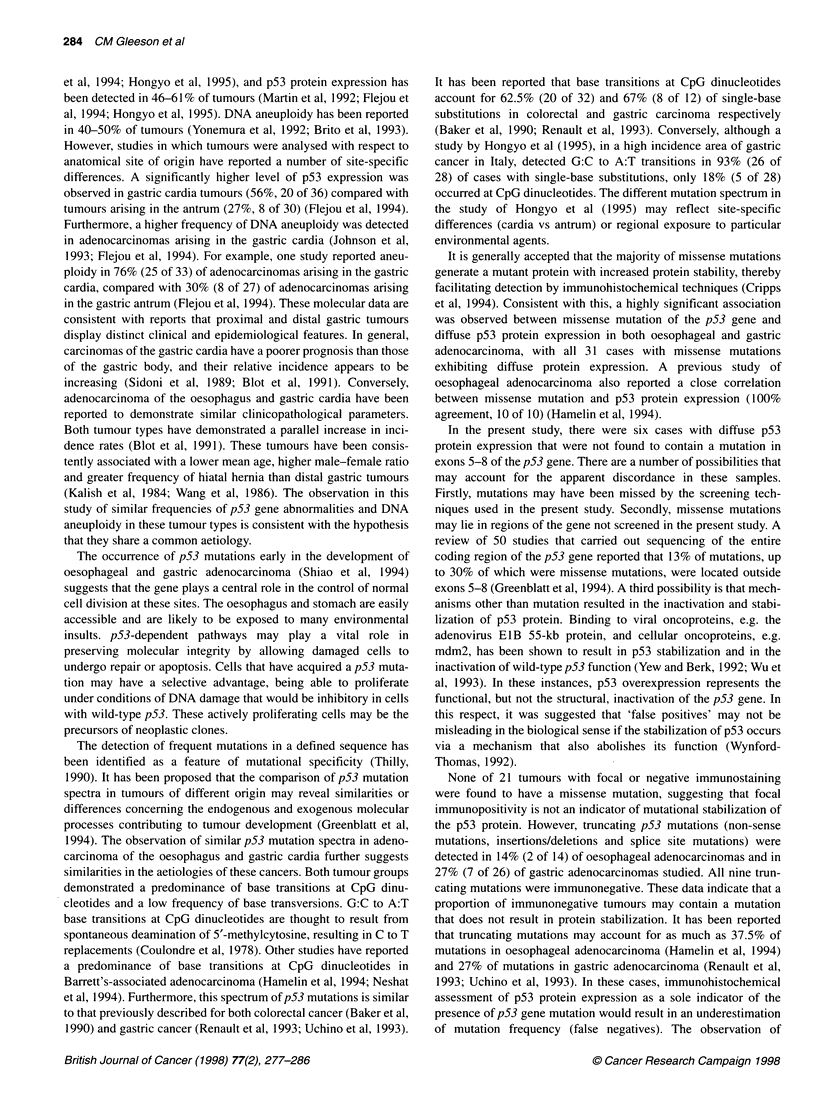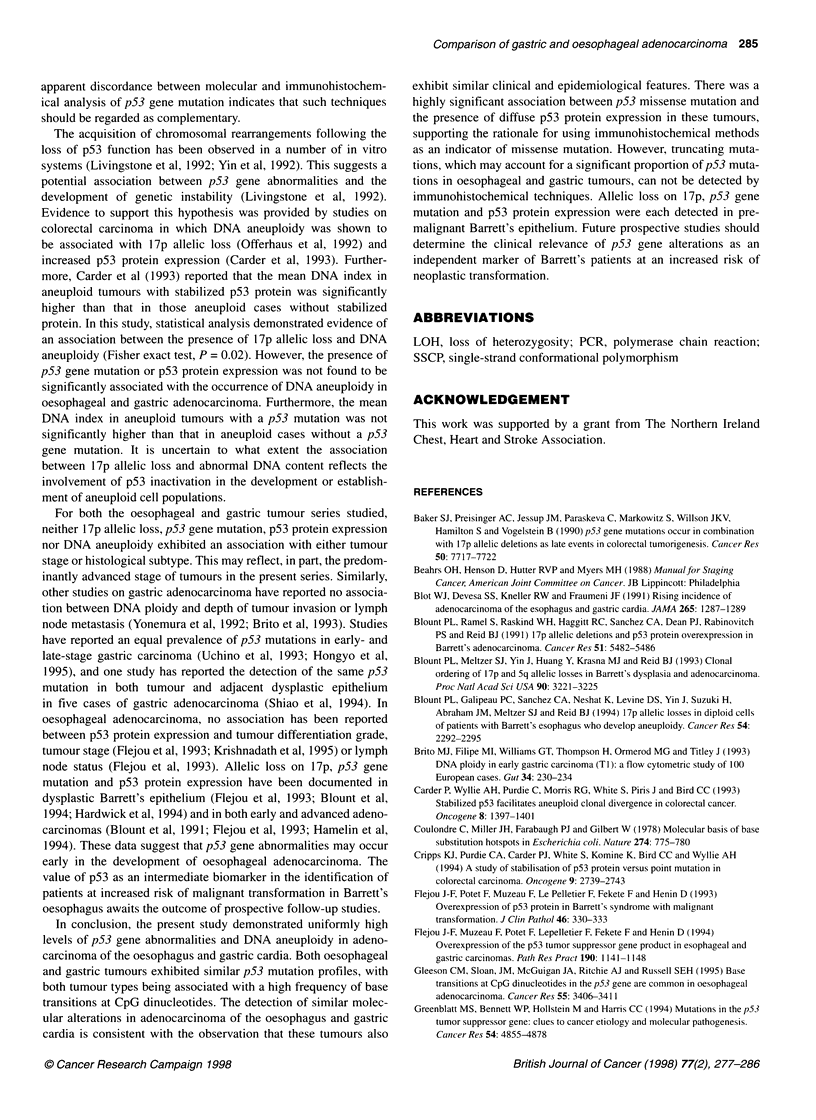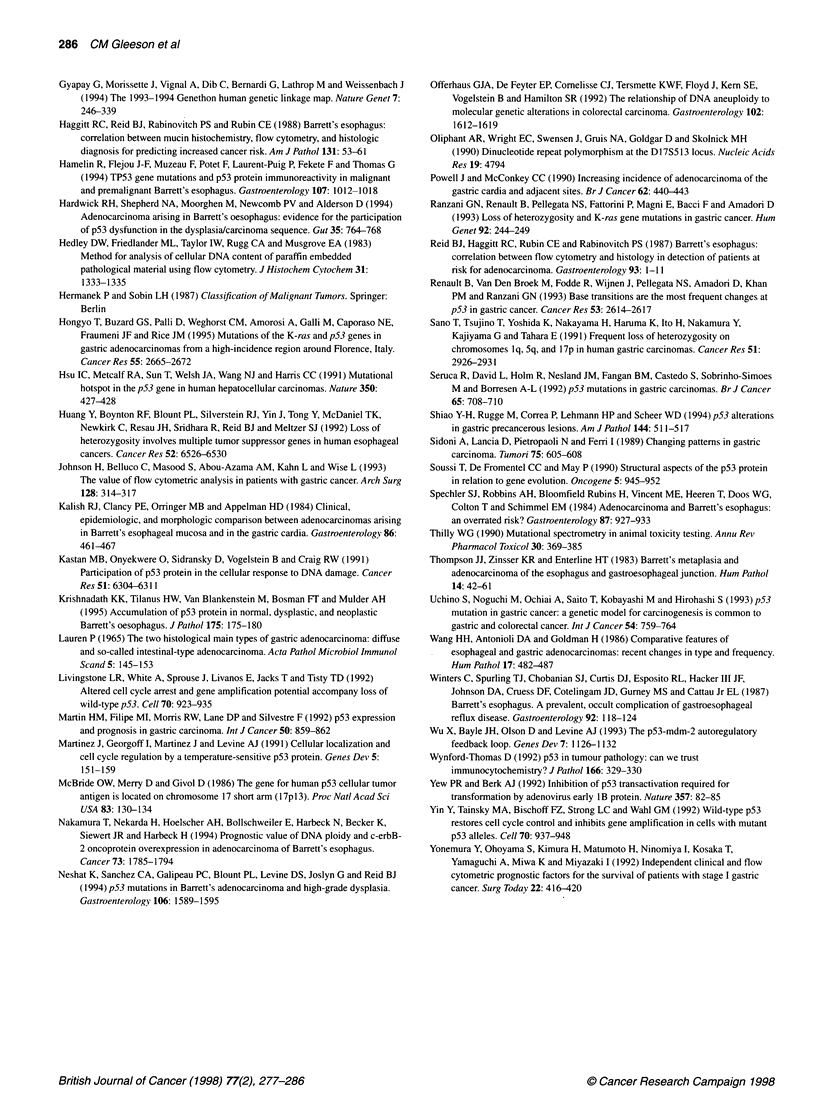# Comparison of p53 and DNA content abnormalities in adenocarcinoma of the oesophagus and gastric cardia.

**DOI:** 10.1038/bjc.1998.44

**Published:** 1998

**Authors:** C. M. Gleeson, J. M. Sloan, D. T. McManus, P. Maxwell, K. Arthur, J. A. McGuigan, A. J. Ritchie, S. E. Russell

**Affiliations:** Department of Medical Genetics, The Queen's University of Belfast, Belfast City Hospital, N Ireland, UK.

## Abstract

**Images:**


British Journal of Cancer (1998) 77(2), 277–286
© 1998 Cancer Research Campaign

### Comparison of *p53* and DNA content abnormalities in adenocarcinoma of the oesophagus and gastric cardia

**CM Gleeson[1], JM Sloan[2], DT McManus[2], P Maxwell[2], K Arthur[2], JA McGuigan[3], AJ Ritchie[3] and SEH Russell[1]**

[1]Department of Medical Genetics, The Queen's University of Belfast, Belfast City Hospital, Lisburn Road, Belfast BT9 7AB; [2]Department of Pathology and [3]The Regional Thoracic Unit, Royal Hospitals Trust, Grosvenor Road, Belfast BT12 6BJ, N Ireland, UK

**Summary** This study examined the association between 17p allelic loss, *p53* gene mutation, p53 protein expression and DNA aneuploidy in a series of adenocarcinomas arising in the oesophagus and gastric cardia. 17p allelic loss was detected in 79% (15 of 19) of oesophageal and in 83% (29 of 35) of gastric adenocarcinomas. *p53* mutations were detected in 70% (14 of 20) and 63% (26 of 41) of oesophageal and of gastric adenocarcinomas respectively. Both tumour types were associated with a predominance of base transitions at CpG dinucleotides. In five cases of oesophageal adenocarcinoma, the same mutation was detected both in tumour and in adjacent dysplastic Barrett's epithelium. Diffuse p53 protein expression was detected in 65% (13 of 20) and 59% (24 of 41) of oesophageal and of gastric tumours, respectively, and was associated with the presence of *p53* missense mutation (Chi-squared, $P < 0.0001$). DNA aneuploidy was detected in 80% (16 of 20) of oesophageal and in 70% (28 of 40) of gastric tumours. No association was found between p53 or DNA content abnormalities and tumour stage or histological subtype. In conclusion, this study detected a similar pattern of p53 alterations in adenocarcinoma of the oesophagus and gastric cardia – molecular data consistent with the observation that these tumours demonstrate similar clinical and epidemiological features.

**Keywords:** adenocarcinoma; oesophagus; gastric cardia; Barrett's oesophagus; *p53*

In recent years, the incidence rates of adenocarcinoma of the oesophagus and gastric cardia have increased steadily, while there has been a decrease in the proportion of tumours arising in the distal stomach (Powell and McConkey, 1990; Blot et al, 1991). Gastric adenocarcinomas represent a heterogeneous group of tumours. Adenocarcinomas arising in the proximal stomach (gastric cardia) were reported to demonstrate biological and epidemiological features distinct from those arising in the distal stomach (gastric antrum) (Sidoni et al, 1989; Blot et al, 1991). Conversely, adenocarcinoma of the gastric cardia was reported to demonstrate clinicopathological features similar to those of oesophageal adenocarcinoma (Kalish et al, 1984; Wang et al, 1986). These data suggest that tumours arising in different anatomical sites in the stomach are associated with distinct aetiologies, while adenocarcinomas arising in the oesophagus and gastric cardia may share similar aetiologies.

The specific aetiological factors underlying the increasing incidence of adenocarcinoma of the oesophagus and gastric cardia remain unresolved. Barrett's oesophagus, a condition in which the squamous epithelium normally lining the lower oesophagus is replaced by a metaplastic columnar epithelium, represents one known risk factor for oesophageal adenocarcinoma. This condition arises in 10–12% of patients with chronic gastro-oesophageal reflux (Winters et al, 1987). The estimated risk of a patient with Barrett's oesophagus developing adenocarcinoma is 30 to 40 times higher than in the general population (Spechler et al, 1984). Tumour development in these patients is proposed to occur via a series of dysplastic cell changes, recognized histologically as a metaplasia–dysplasia–carcinoma sequence (Thompson et al, 1983).

*Received 17 February 1997*
*Revised 13 May 1997*
*Accepted 26 June 1997*

*Correspondence to:* SEH Russell

The *p53* gene, localized to chromosome 17p13 (McBride et al, 1986), encodes a 53-kDa nuclear phosphoprotein that functions in a signal transduction pathway, causing an arrest of cells in the $G_1$ phase of the cell cycle in response to DNA damage (Kastan et al, 1991). Allelic loss on chromosome 17p and *p53* gene mutations are among the most common genetic abnormalities documented in human cancers (Greenblatt et al, 1994). The majority of *p53* mutations have been reported to occur in exons 5–8, corresponding to the evolutionarily conserved domains of the protein (Soussi et al, 1990; Greenblatt et al, 1994). Mutations in these domains were found to be associated with loss of wild-type p53 function, including growth suppression (Martinez et al, 1991). Inactivation of *p53* was reported to be associated with the development of genomic instability and DNA aneuploidy (Livingstone et al, 1992; Yin et al, 1992; Carder et al, 1993).

p53 and DNA content abnormalities have been implicated in the development of both oesophageal and gastric adenocarcinoma (Reid et al, 1987; Yonemura et al, 1992; Blount et al, 1993; Renault et al, 1993; Hamelin et al, 1994). However, molecular studies in which gastric tumours were analysed with respect to anatomical site of origin (cardia vs antrum) have reported a number of site-specific differences. Significantly higher levels of p53 protein expression (Flejou et al, 1994) and DNA aneuploidy (Johnson et al, 1993; Flejou et al, 1994) have been reported in gastric cardia tumours compared with tumours arising in the gastric antrum. These molecular data are consistent with the observation that proximal and distal gastric tumours display distinct epidemiological features (Blot et al, 1991). Conversely, adenocarcinoma of the oesophagus and gastric cardia demonstrate similar clinical and epidemiological features (Wang et al, 1986; Blot et al, 1991). The aim of the present study was, firstly, to document the pattern of p53 and DNA content abnormalities in a homogenous series of adenocarcinomas arising in the gastric cardia and to compare the pattern with that detected in a series of oesophageal adenocarcinomas.

**Table 1** PCR amplification and sequencing primers for *p53*, exons 5–8

| *p53* | Primer sequences | Application: PCR and/or sequencing[a] |
|---|---|---|
| Exon 5 | 5′-TGTTCACTTGTGCCCTGACT-3′ | Left PCR and sequencing primer |
| | 5′-AGCAATCAGTGAGGAATCAG-3′ | Right PCR and sequencing primer |
| Exon 6 | 5′-TGGTTGCCCAGGGTCCCCAG-3′ | Left PCR and sequencing primer |
| | 5′-GGAGGGCCACTGACAACCA-3′ | Right PCR and sequencing primer |
| Exon 7 | 5′-CTTGCCACAGGTCTCCCCAA-3′ | Left PCR and sequencing primer |
| | 5′-AGGGGTCAGCGGCAAGCAGA-3′ | Right external PCR primer |
| | 5′-TGTGCAGGGTGGCAAGTGGC-3′ | Right internal sequencing primer |
| Exon 8 | 5′-TTCCTTACTGCCTCTTGCTT-3′ | Left PCR and sequencing primer |
| | 5′-AGGCATAACTGCACCCTTGG-3′ | Right PCR and sequencing primer |

[a]Sequence analysis of exons 5, 6 and 8 used the same primers as those used for PCR amplification. For exon 7, an internal primer was used in the reverse direction to give optimal sequencing profiles.

**Table 2** 17p allelic loss, *p53* gene mutation, p53 protein expression and DNA content in Barrett's epithelium and oesophageal adenocarcinoma

| | | | LOH at 17p13[c] | | | *p53* Mutation analysis | | | | | | |
|---|---|---|---|---|---|---|---|---|---|---|---|---|
| Patient no. | Stage[a] | Histology[b] | D17S 513 | D17S796 | SSCP[d] | Codon | Nucleotide change | Nature of nucleotide substitution | Amino acid change | ICC[g] | DNA content | DI[h] |
| 104 | I | W/MD | a | H | 6 | 196 | CGA → TGA | G:C to A:T[e] | Arg → Stop | –ve | Aneuploid | 1.3 |
| 108 | I | MD | b | H | 6 | 212 | 2-bp Deletion | | Frameshift | –ve | Aneuploid | 1.5 |
| | | HGD | – | H | 6 | 212 | 2-bp Deletion | | Frameshift | –ve | – | |
| | | LGD | – | H | –ve | | | | | | – | |
| | | im | – | H | –ve | | | | | | – | |
| 90 | IIA | WD | H | a | 8 | 273 | CGT → CAT | G:C to A:T[e] | Arg → His | + + + | Aneuploid | 3.2 |
| 36 | IIB | WD | H | – | –ve | | | | | + | Aneuploid | 1.8 |
| 88 | IIA | W/MD | b | H | 8 | 282 | CGG → TGG | G:C to A:T[e] | Arg → Trp | + + + | Aneuploid | 1.8 |
| 110 | IIA | MD | ab | H | –ve | | | | | –ve | Diploid | 1.0 |
| 112 | IIA | MD | b | a | 5 | 158 | CGC → CTC | G:C to T:A | Arg → Leu | + + + | Aneuploid | 1.4 |
| 125 | IIA | MD | b | b | –ve | | | | | –ve | Aneuploid | 1.6 |
| | | HGD | – | b | –ve | | | | | –ve | – | |
| 76 | IIB | MD | a | H | 8 | 282 | CGG → TGG | G:C to A:T[e] | Arg → Trp | + + + | Aneuploid | 1.5 |
| | | HGD | – | H | 8 | 282 | CGG → TGG | G:C to A:T[e] | Arg → Trp | + + + | – | |
| 22 | IIA | PD | b | – | 7 | 248 | CGG → TGG | G:C to A:T[e] | Arg → Trp | + + + | Aneuploid | 1.3 |
| | | HGD | – | – | 7 | 248 | CGG → TGG | G:C to A:T[e] | Arg → Trp | + + + | – | |
| 120 | IIA | PD | a | – | 5 | N.D. | | | | + + + | Aneuploid | 2.3 |
| 123 | IIA | PD | ab | ab | 8 | 266 | GGA → GAA | G:C to A:T | Gly → Glu | + + + | Diploid | 1.0 |
| 109 | II | PD | b | – | 5 | 175 | CGC → CAC | G:C to A:T[e] | Arg → His | + + + | Diploid | 1.0 |
| 54 | III | MD | ab | H | –ve | | | | | + | Diploid | 1.0 |
| 99 | III | MD | H | b | 7 | 248 | CGG → TGG | G:C to A:T[e] | Arg → Trp | + + + | Aneuploid | 1.4 |
| 124 | III | MD | H | b | 8 | 285 | GAG → AAG | G:C to A:T | Glu → Lys | + + + | Aneuploid | 1.4 |
| | | HGD | – | b | 8 | 285 | GAG → AAG | G:C to A:T | Glu → Lys | + + + | – | |
| 29 | III | M/PD | ab | ab | 5 | 175 | CGC → CAC | G:C to A:T[e] | Arg → His | + + + | Aneuploid | 1.4 |
| | | HGD | – | ab | 5 | 175 | CGC → CAC | G:C to A:T[e] | Arg → His | + + + | – | |
| | | LGD | – | ab | 5 | 175 | CGC → CAC | G:C to A:T[e] | Arg → His | + + + | – | |
| | | im | – | ab | –ve | | | | | –ve | – | |
| 50 | III | PD | H | b | 7 | 242 | TGC → TTC | G:C to T:A | Cys → Phe | + + + | Aneuploid | 1.7 |
| 74 | III | PD | b | b | 7 | 248 | CGG → CAG | G:C to A:T[e] | Arg → Gln | + + + | Aneuploid | 1.6 |
| 43* | III | PD | b | b | –ve | | | | | –ve | Aneuploid | 1.7 |

[a]TNM – stage I: 1, 0, 0; stage IIA: 2, 0, 0/3, 0, 0; stage IIB: 1, 1, 0/2, 1, 0; stage III: 3, 1, 0/4, 0, 0. [b]The tumours were classified according to the procedure of Lauren (1965). *Tumour no. 43 was a diffuse-type adenocarcinoma. All other tumours were intestinal-type adenocarcinoma arising on a background of Barrett's metaplasia. WD, well differentiated; MD, moderately differentiated; PD, poorly differentiated; HGD, high-grade dysplasia; LGD, low-grade dysplasia; im, intestinal metaplasia. [c]a, Upper allele retained; b, lower allele retained; ab, heterozygous with no loss; H, homozygous; –, not determined. [d]Exon exhibiting band shift. –ve, band shift not detected. [e]G:C to A:T base transitions occurring at CpG dinucleotides. [f]Predicted change in amino acid sequence as a result of mutation. [g]ICC, immunohistochemistry; +, < 10% positive cells, focal staining pattern; ++, 10–50% positive cells; +++, > 50% positive cells, diffuse staining pattern; –ve, negative. [h]DI, DNA index.

© Cancer Research Campaign 1998

**Table 3** 17p allelic loss, *p53* gene mutation, p53 protein expression and DNA content in gastric adenocarcinoma

| | | | LOH at 17p13[c] | | | | *p53* mutation analysis | | | | | |
|---|---|---|---|---|---|---|---|---|---|---|---|---|
| Patient no. | Stage[a] | Histology[b] | D17S 513 | D17S796 | SSCP[d] | Codon | Nucleotide change | Nature of nucleotide substitution | Amino acid change[f] | ICC[g] | DNA content | DI[h] |
| 105 | IIA | I, WD | a | H | 8 | 273 | CGT → TGT | G:C to A:T[e] | Arg → Cys | + + + | Diploid | 1.0 |
| 37 | IIA | I, WD | b | b | 6 | 215 | AGT → AGG | A:T to C:G | Ser → Arg | + + + | Aneuploid | 1.6 |
| 52 | IIA | I, WD | H | ab | –ve | | | | | + + + | Diploid | 1.0 |
| 72 | IIA | I, MD | a | – | –ve | | | | | –ve | Aneuploid | 1.6 |
| 121 | IIA | I, MD | a | a | 6 | 193 | CAT → CTT | A:T to T:A | His → Leu | + + + | Aneuploid | 1.5 |
| 95 | IIA | I, MD | H | H | 8 | 306 | CGA → TGA | G:C to A:T[e] | Arg → Stop | –ve | Aneuploid | 1.6 |
| 31 | IIA | I, MD | H | b | 8 | 282 | CGG → TGG | G:C to A:T[e] | Arg → Trp | + + + | Diploid | 1.0 |
| 30 | IIA | I, MD | b | – | –ve | | | | | + + | Diploid | 1.0 |
| 92 | IIB | I, MD | b | b | 7 | 248 | CGG → CAG | G:C to A:T[e] | Arg → Gln | + + + | Aneuploid | 1.2 |
| 85 | IIB | I, PD | a | a | 5 | 175 | CGC → CAC | G:C to A:T[e] | Arg → His | + + + | Diploid | 1.0 |
| | | | | | 6(C + T) | 213 | CGA → CGG | | Arg → Arg | | | |
| 98 | IIA | D, PD | H | H | 5 | 146 | TGG → TGA | G:C to A:T | Trp → Stop | –ve | Aneuploid | 1.5 |
| 48 | III | I, WD | H | a | –ve | | | | | + | Aneuploid | 1.2 |
| 21 | III | I, WD | – | b | –ve | | | | | + + + | Aneuploid | 1.6 |
| 24 | III | I, WD | – | a | 5 | 173 | GTG → TTG | G:C to T:A | Val → Leu | + + + | Aneuploid | 1.4 |
| 83 | III | I, MD | a | – | 5 | 173 | GTG → ATG | G:C to A:T | Val → Met | + + + | Aneuploid | 1.4 |
| 111 | III | I, MD | b | a | 5 | 175 | CGC → CAC | G:C to A:T[e] | Arg → His | + + + | Aneuploid | 1.4 |
| 68 | III | I, MD | H | ab | –ve | | | | | + + | Aneuploid | 1.8 |
| 77 | III | I, MD | ab | – | –ve | | | | | + | Diploid | 1.0 |
| 75 | III | I, MD | H | – | –ve | | | | | –ve | Aneuploid | 1.4 |
| 97 | III | I, PD | H | ab | –ve | | | | | + | Aneuploid | 1.3 |
| 35 | III | I, PD | b | H | –ve | | | | | + + + | Aneuploid | 2.6 |
| 103 | III | I, PD | – | b | 7 | 249 | AGG → ATG | G:C to T:A | Arg → Met | + + + | Aneuploid | 1.6 |
| 127 | III | I, PD | a | a | 7 | 245 | GGC → AGC | G:C to A:T[e] | Gly → Ser | + + + | Diploid | 1.0 |
| 107 | III | I, PD | b | b | 5 | 138 | GCC → GTC | G:C to A:T | Ala → Val | + + + | Aneuploid | 1.6 |
| 115 | III | I, PD | – | – | 8 | 273 | CGT → CAT | G:C to A:T[e] | Arg → His | + + + | Aneuploid | 1.3 |
| 44 | III | I, PD | H | ab | –ve | | | | | +/+ + | Diploid | 1.0 |
| 94 | III | I, PD | ab | a | –ve | | | | | + | Aneuploid | 1.8 |
| 100 | III | I, PD | H | b | 8 | 282 | CGG → TGG | G:C to A:T[e] | Arg → Trp | + + + | Diploid | 1.0 |
| 122 | III | I, PD | H | b | 8 | 273 | CGT → CAT | G:C to A:T[e] | Arg → His | + + + | Aneuploid | 2 |
| 40 | III | I, PD | b | – | –ve | | | | | + + + | Aneuploid | 1.2 |
| 69 | III | I, PD | ab | – | 8 | 306 | CGA → TGA | G:C to A:T[e] | Arg → Stop | –ve | No result | – |
| 89 | III | I, PD | a | – | 8 | Sds | gt → at | | | –ve | Aneuploid | 1.4 |
| 93 | III | I, PD | – | – | –ve | | | | | –ve | Aneuploid | 1.2 |
| 67 | III | D, PD | H | a | 5 | 168 | CAC → CGC | A:T to G:C | His → Arg | + + + | Aneuploid | 1.6 |
| 102 | III | D, PD | H | a | –ve | | | | | + + + | Diploid | 1.0 |
| 84 | III | D, PD | H | b | 6 | 222 | 9-bp Deletion | | | + | Aneuploid | 1.9 |
| 38 | III | D, PD | b | b | 8 | 266 | GGA → GAA | G:C to A:T | Gly → Glu | + + + | Aneuploid | 2.0 |
| 116 | III | D, PD | H | H | 7 | 245 | GGC → GAC | G:C to A:T | Gly → Asp | + + + | Diploid | 1.0 |
| 81 | III | D, PD | H | b | 7 | Sds | gt → tt | | | –ve | Diploid | 1.0 |
| 58 | III | D, PD | b | a | 6 | Sds | gt → at | | | –ve | Aneuploid | 1.3 |
| 101 | III | D, PD | a | b | 8 | 273 | CGT → TGT | G:C to A:T[e] | Arg → Cys | + + + | Aneuploid | 1.5 |

[a]TNM – stage IIA: 2, 0, 0/3, 0, 0; stage IIB: 1, 1, 0/2, 1, 0; Stage III: 3, 1, 0/4, 0, 0. [b]The tumours were classified according to the procedure of Lauren (1965). I, intestinal type; D, diffuse type; WD, well differentiated; MD, moderately differentiated; PD, poorly differentiated. [c]a, Upper allele retained; b, lower allele retained; ab, heterozygous with no loss; H, homozygous; –, not determined. [d]Exon exhibiting band shift. –ve, band shift not detected. Sds, splice donor site. [e]G:C to A:T base transitions occurring at CpG dinucleotides. [f]Predicted change in amino acid sequence as a result of mutation. [g]ICC, immunohistochemistry; +, < 10% positive cells, focal staining pattern; ++, 10–50% positive cells; +++, > 50% positive cells, diffuse staining pattern; –ve, negative. [h]DI, DNA index.

Secondly, a number of studies have implicated p53 inactivation in the malignant transformation of Barrett's oesophagus (Hamelin et al, 1994; Neshat et al, 1994; Gleeson et al, 1995). In order to confirm and extend these observations, this study assessed the involvement of p53 in premalignant Barrett's epithelium.

### MATERIALS AND METHODS

### Tissue collection and DNA extraction

Matched normal and tumour tissue samples were obtained intra-operatively from 20 patients with Barrett's-associated oesophageal adenocarcinoma and from 41 patients with adenocarcinoma of the gastric cardia. Barrett's-associated adenocarcinomas were categorized as those tumours with histological evidence of having arisen against a background of Barrett's epithelium. All oesophageal cases were associated with Barrett's metaplasia extending greater than 3 cm into the oesophagus, and all tumours exhibited predominant localization in the oesophagus. The gastric cardia tumours were located near the gastro-oesophageal junction. None had associated Barrett's epithelium. In six cases of Barrett's-associated adenocarcinoma, residual Barrett's epithelium adjacent to adenocarcinoma was obtained for analysis. The tissue was snap frozen in liquid nitrogen and stored at –70°C. The tissue samples were histologically assessed

© Cancer Research Campaign 1998

**Figure 1** Representative examples of allelic loss at the D17S796 locus on chromosome 17p13. (1) Case no. 50; (2) case no. 90; (3) case no. 99; (4) case no. 112; (5) case no. 21; (6) case no. 31; (7) case no. 94; (8) case no. 100; (9) case no. 101; (10) case no. 103; (11) case no. 122; (12) case no. 127. C, control DNA; T, tumour DNA

**Figure 2** Diagrammatic representation of the *p53* mutation spectrum detected in adenocarcinoma of the oesophagus and gastric cardia. Other, insertions/deletions/splice site mutations

**Figure 3** Automated DNA sequence analysis of *p53*, exon 5 in patient no. 29. Wild-type *p53* sequence was detected in (**A**) control DNA and (**B**) non-dysplastic intestinal metaplasia. A C to T substitution (antisense strand), resulting in an arginine to histidine substitution, was detected at codon 175 in (**C**) low-grade dysplasia, (**D**) high-grade dysplasia and (**E**) tumour. The mutant allele is indicated by an arrow by a consultant pathologist (JMS) and classified according to the procedure of Lauren (1965). Tumour stage was assessed using the American Joint Committee on Cancer (A.J.C.C.) and the International Union against Cancer (U.I.C.C.) criteria for pathological staging (Hermanek and Sobin, 1987; Beahrs et al, 1988). Cryostat sectioning was carried out to select tumour-rich tissue for analysis, and microdissection was used to isolate all preneoplastic lesions. DNA was extracted from matched normal and tumour tissue samples as described previously (Gleeson et al, 1995).

### Allelic loss analysis

To detect 17p allelic loss in the tumour tissue, two dinucleotide repeat polymorphisms, D17S513 and D17S796, mapping to 17p13 were analysed (Oliphant et al, 1990; Gyapay et al, 1994). Polymerase chain reaction (PCR) amplification and microsatellite analysis were carried out as described previously (Gleeson et al, 1995). LOH was scored by direct visual comparison of the relative allelic ratios present in matched normal and tumour DNAs. Allele loss was scored if one allele was absent or exhibited altered signal intensity in tumour DNA relative to the allelic ratio of normal DNA.

### Mutational analysis of *p53*, exons 5–8

For PCR amplification of *p53*, exons 5–8, primers homologous to sequences in the adjacent introns were used (Hsu et al, 1991, Table 1). Genomic DNA (300 ng) was incubated in a total volume of 100 μl of PCR buffer [50 mM potassium chloride, 10 mM Tris-HCl, pH 9.0, 1 mM magnesium chloride (1.5 mM for exons 5 and 8), 0.1% Triton X-100] containing 0.25 μM of each primer, 0.2 mM dNTPs and 5 units of *Taq* polymerase (Promega). There was an initial denaturation step at 94°C for 5 min, followed by 35 cycles of 1 min at 94°C, 1 min at 56°C (62°C for exon 6) and 30 s at 72°C, with a final extension step of 72°C for 5 min.

A number of *p53* mutations ($n$ = 19) were initially identified by direct DNA sequencing on a subgroup of 35 tumours. Sequence analysis was carried out as described previously (Gleeson et al, 1995). These 19 control mutations were analysed to select optimal gel conditions for mutation detection by non-isotopic single-strand conformational polymorphism (SSCP). For each exon, one set of gel conditions was identified that detected all known sequence variants in that exon. All exons were analysed on 1-mm-thick 8% polyacrylamide gels with 2.6% cross-linking. The optimal conditions selected were 4°C without glycerol (exon 6), 24°C without glycerol (exon 5) and 24°C with 5% glycerol (exons 7 and 8).

© Cancer Research Campaign 1998

**Table 4** Association between *p53* gene mutation and 17p allelic loss in oesophageal and gastric adenocarcinoma

| | 17p Allelic loss | | |
|---|---|---|---|
| ***p53* Gene mutation** | **LOH** | **No LOH** | **Fisher exact test** |
| Mutation | 33 | 3 | |
| No mutation | 11 | 7 | $P = 0.02$ |

**Table 5** Association between *p53* gene mutation and p53 protein expression in oesophageal and gastric adenocarcinoma

| | p53 Protein expression | | |
|---|---|---|---|
| ***p53* Gene mutation** | **Diffuse** | **Focal/negative** | **Chi-squared** |
| Mutation | 31 | 9 | |
| No mutation | 6 | 12 | $P = 0.003$ |

**Table 6** Association between *p53* missense mutation and p53 protein expression in oesophageal and gastric adenocarcinoma

| | p53 Protein expression | | |
|---|---|---|---|
| ***p53* Missense mutation** | **Diffuse** | **Focal/negative** | **Chi-squared** |
| Missense mutation | 31 | 0 | |
| No missense mutation | 6 | 21 | $P < 0.0001$ |

Aliquots of PCR product (5 µl) were denatured in alkali buffer (10× stock: 500 mM sodium hydroxide, 10 mM EDTA) at 42°C for 30 min and immediately placed on ice. Loading buffer [10× stock: 0.5% (w/v) bromophenol blue, 0.5% (w/v) xylene cyanol in deionized formamide] was added and the samples were loaded to the appropriate gel. Electrophoresis was performed at 400 V for 3–6 h using 1 × TBE buffer. Electrophoresis was carried out using a Protean II vertical electrophoresis tank (Biorad), which was modified to allow buffer recirculation through a closed-circuit loop placed in a temperature-regulated water bath, allowing accurate and reproducible temperature control. After electrophoresis, the gel was fixed in an aqueous solution of 10% (v/v) ethanol and 0.5% (v/v) acetic acid for 3 min (×2). The gel was stained in an aqueous solution of 0.1% (w/v) silver nitrate for 15 min and visualized by immersion in an aqueous solution of 0.1% (w/v) sodium hydroxide and 0.1% (v/v) formaldehyde for 20 min. The gel was fixed for 10 min in an aqueous solution of 0.75% (w/v) sodium carbonate. Band shifts were identified by direct comparison of the pattern of single-stranded bands in control and tumour DNA from the same individual. SSCP analysis, using the optimized gel conditions, was used to screen for *p53* mutations in the remaining 26 cases. Sequence analysis was used to confirm and identify the mutation present in each sample exhibiting an altered mobility shift.

### Immunohistochemistry

Paraffin sections (4 µm) were dewaxed in xylene, rehydrated through descending grades of alcohol and immersed in 3% (v/v) alcoholic hydrogen peroxide for 10 min. The sections were immersed in 0.1 M sodium citrate buffer, pH 6.0, and microwaved for 30 min. The sections were stained with the DO7 antibody (Novocastra) at an optimal dilution of 1:50 for 30 min at room temperature and then rinsed in TBS to remove unbound primary antiserum. A biotinylated anti-mouse secondary antiserum (Dako) was incubated at an optimal dilution (1:200 in 1:25 normal human serum) for 30 min at room temperature. The sections were rinsed in TBS and the bound antiserum was visualized using the streptavidin–biotin complex immunoperoxidase protocol, using diaminobenzidine as a chromogen (Dako, UK). Sections were counterstained in Harris's haematoxylin and mounted with DPX mounting medium (Diachem). Sections demonstrating nuclear staining in greater than 50% of tumour cells were categorized as diffuse staining, while sections demonstrating nuclear staining in less than 10% of tumour cells were categorized as focal staining. A known strongly staining case was used as a positive control. Omission of the primary antiserum served as a negative control.

### Flow cytometry

Paraffin sections (50 µm) were processed for flow cytometry using a modification of the method described by Hedley et al (1983). Nuclear suspensions were stained with propidium iodide solution [propidium iodide (3 mg per 100 ml of phosphate-buffered saline) containing 10 mg of Ribonuclease A and 0.5 ml of 0.5% Triton X-100] at 4°C for 30 min. Samples were analysed on a Coulter Epics Elite Flow Cytometer equipped with a 15-mW Argon laser, excitation beam 488 nm. The DNA histogram upon which analysis was performed in each case was gated on Forward Angle Light Scatter (FALS) to minimize doublets and clumps, but included a proportion of debris. The histograms were analysed using Multicycle Software (Phoenix Flow Systems). This analysis allowed debris and sliced nuclei to be accounted for and to be subtracted from the histograms. Histograms that showed a single $G_0G_1$ peak with a corresponding $G_2$ + M peak were classified as DNA diploid. Diploid histograms were only accepted if the coefficient of variation (CV) at half the peak height was less than 10%. DNA aneuploidy was defined as the presence of an additional $G_0G_1$ peak that contained > 10% of the total cell population. The DNA index, defined as the modal DNA content of the aneuploid cell population divided by the modal DNA content of the diploid cell population, was calculated for each aneuploid histogram.

### RESULTS

The mean age at diagnosis with oesophageal adenocarcinoma was 61 years (range 31–75 years), and the patient group demonstrated a male–female ratio of 17:3. The tumour series included two stage I tumours, 11 stage II tumours and seven stage III tumours. With respect to histological subtype, the tumours included 19 cases of the intestinal type (well–moderately differentiated, $n = 12$; poorly differentiated, $n = 7$) and one poorly differentiated diffuse-type adenocarcinoma (Table 2). The mean age at diagnosis with adenocarcinoma of the gastric cardia was 63 years (range 44–80 years), and the patient group exhibited a male–female ratio of 31:10. The tumour group included 11 stage II tumours and 30 stage III tumours. Histological assessment identified 32 gastric adenocarcinomas of the intestinal type (well–moderately differentiated, $n = 17$; poorly differentiated, $n = 15$) and nine gastric adenocarcinomas of the diffuse type (Table 3).

Allelic loss on chromosome 17p was investigated using two highly informative dinucleotide repeat polymorphisms mapping to

© Cancer Research Campaign 1998

Figure 4 Representative examples of immunostaining patterns obtained with the DO7 antiserum. (**A**) +, Focal staining of individual cell nuclei in dysplastic Barrett's epithelium (original magnification × 400); (**B**) +++, diffuse immunostaining of a moderately differentiated gastric adenocarcinoma, case no. 92 (original magnification × 250); (**C**) +++, diffuse immunostaining in dysplastic Barrett's epithelium, case no. 29 (original magnification × 100). Intervening non-malignant stroma is immunonegative chromosome 17p13. For oesophageal adenocarcinoma, 95% (19 of 20) of samples were informative at either one or both of the polymorphic loci analysed, and allelic loss was detected in 79% (15 of 19) of informative cases (Table 2). Allelic loss was detected in 83% (29 of 35) of informative cases of gastric adenocarcinoma (Table 3). With the exception of case no. 94, allele loss scores were concordant in all cases for which both loci were informative. Representative examples of allelic loss at the D17S796 microsatellite are shown in Figure 1.

Mutational analysis of *p53*, exons 5–8, resulted in the detection of mutations in 70% (14 of 20) of oesophageal adenocarcinomas (Table 2), eleven of which have been documented previously (Gleeson et al, 1995). These included 12 missense mutations, one non-sense mutation and one frameshift mutation, the last being a 2-bp deletion in exon 6. Eighty-five per cent (11 of 13) of the single-base substitutions were G:C to A:T base transitions, with 69% (9 of 13) occurring at CpG dinucleotides. The remaining two single-base changes were G:C to T:A base transversions. Mutations were detected in 63% (26 of 41) of gastric adenocarcinomas (Table 3). These included 19 missense mutations, three non-sense mutations, three splice site mutations and one deletion. Of the 22 single-base substitutions in coding sequence, 82% (18 of 22) were base transitions (17 G:C to A:T, one A:T to G:C), with 55% (12 of 22) occurring at CpG dinucleotides. The remaining four single-base substitutions were base transversions (two G:C to T:A, one A:T to T:A, one A:T to C:G). A graphical representation of the mutational profiles detected in oesophageal and gastric adenocarcinoma is shown in Figure 2.

Premalignant Barrett's epithelium adjacent to oesophageal adenocarcinoma was available for analysis in six cases (nos. 22, 29, 76, 108, 124 and 125). Sequence analysis did not detect a mutation in case no. 125. In the remaining five cases, the same *p53* mutation was detected both in high-grade dysplasia and in adjacent tumour tissue. In case no. 29, the same mutation was also detected in low-grade dysplasia but not in Barrett's intestinal metaplasia (Figure 3). In case no. 108, low-grade dysplasia and Barrett's intestinal metaplasia, negative for dysplasia, were not found to contain the *p53* mutation detected in the adjacent high-grade dysplasia and tumour (Table 2).

Overall, 33 tumours with 17p allelic loss were found to contain a *p53* mutation. A further 11 cases exhibited 17p allelic loss, however mutations were not detected in exons 5–8 of the *p53* gene. *p53* mutations were detected in three cases with retention of heterozygosity on 17p and in four non-informative cases. Mutations were not detected in seven cases that retained heterozygosity on 17p nor in three non-informative cases. Statistical analysis revealed an association between 17p allelic loss and *p53* gene mutation (Table 4, Fisher exact test, $P = 0.02$).

p53 protein expression was assessed using the DO7 antiserum, and representative examples of the p53 immunostaining patterns observed are shown in Figure 4. Weak cytoplasmic staining was detected in focal cells in the basal layers of adjacent gastric mucosa, squamous epithelium of the oesophagus and non-dysplastic Barrett's epithelium. This staining was readily distinguished from the specific p53 nuclear immunostaining detected in dysplastic epithelium and carcinoma. Omission of the primary antiserum resulted in complete abolition of nuclear immunostaining. Diffuse p53 protein expression was detected in 65% (13 of 20) of oesophageal adenocarcinomas and in 59% (24 of 41) of gastric adenocarcinomas. A further 21 cases exhibited focal staining or negative p53 immunoreactivity. The remaining three cases demonstrated immunoreactivity in 10–50% of tumour cells. The staining pattern in these cases was not easily classified as either diffuse (Figure 4B) or focal (Figure 4A). The significance of p53 immunoreactivity in these cases is uncertain, and they were excluded from subsequent statistical analyses. There was a significant association between diffuse p53 protein expression and *p53* gene mutation (Table 5; Chi-squared, $P = 0.003$). In particular, all

© Cancer Research Campaign 1998

31 missense mutations exhibited diffuse p53 protein expression, while nine non-sense or truncating mutations were immunonegative. Statistical analysis revealed a highly significant association between diffuse p53 protein expression and *p53* missense mutation in these tumours (Table 6; Chi-squared, $P < 0.0001$).

There were six cases with premalignant Barrett's epithelium adjacent to carcinoma, and in all cases the immunostaining pattern in the dysplastic Barrett's epithelium was concordant with that detected in the tumour tissue. Two cases (no. 108 and no. 125) showed no immunostaining in either Barrett's epithelium or in adjacent carcinoma. In the remaining four cases (nos. 22, 29, 76 and 124) diffuse p53 expression was detected in both premalignant and malignant lesions; in each of these cases, sequence analysis confirmed the presence of a missense mutation in both dysplastic and invasive lesions (Table 2). Figure 4C shows an example of diffuse p53 protein expression detected in dysplastic Barrett's epithelium and in adjacent carcinoma.

DNA aneuploidy was detected in 80% (16 of 20) of oesophageal tumours (Table 2) and in 70% (28 of 40) of gastric tumours (Table 3). Figure 5 shows representative examples of diploid and aneuploid flow cytometric profiles. Allelic loss on 17p was associated with the occurrence of DNA aneuploidy (Fisher exact test, $P$ = 0.02). There was no association between DNA aneuploidy and *p53* gene mutation or p53 protein expression.

There was no significant association between *p53* gene abnormalities or DNA content abnormalities and tumour stage or histological subtype in either oesophageal or gastric adenocarcinoma.

### DISCUSSION

The present study investigated the association between 17p allelic loss, *p53* gene mutation and p53 protein expression in a series of adenocarcinomas arising in the oesophagus and gastric cardia. The association between *p53* gene abnormalities and DNA content in these tumours was also assessed.

Uniformly high levels of 17p allelic loss, *p53* gene mutation, p53 protein expression and DNA aneuploidy were detected in both adenocarcinoma of the oesophagus and gastric cardia. Similarly, previous studies have documented 17p allelic loss in 69–100% of oesophageal adenocarcinomas (Blount et al, 1991, 1993; Huang et al, 1992). *p53* gene mutations were reported in 55% (6 of 11) and 88% (15 of 17) of oesophageal adenocarcinomas (Hamelin et al, 1994; Neshat et al, 1994), while p53 protein expression was detected in 53–87% of cases (Flejou et al, 1993; Hamelin et al, 1994; Hardwick et al, 1994). The presence of aneuploidy in Barrett's oesophagus was reported to occur with increasing frequency during the histological progression from metaplasia to dysplasia and carcinoma (Reid et al, 1987; Haggitt et al, 1988). Studies have consistently documented high levels of DNA aneuploidy in oesophageal adenocarcinoma, typically ranging from 80% to 100% of cases (Reid et al, 1987; Nakamura et al, 1994).

A number of studies have reported a close association between p53 overexpression in adenocarcinoma and in adjacent highly dysplastic Barrett's epithelium (Flejou et al, 1993; Hardwick et al, 1994). Consistent with these observations, the present study identified five cases in which the same *p53* gene mutation was present in high-grade dysplasia and in adjacent carcinoma, suggesting that *p53* mutation preceded the development of invasive carcinoma in these patients. Furthermore, in one of these cases (no. 29), the same *p53* gene mutation was also detected in coexisting low-grade dysplasia, but not in adjacent non-dysplastic Barrett's epithelium.

**Figure 5** Representative DNA histograms of (**A**) a diploid tumour cell population (case no. 109) and (**B**) an aneuploid tumour cell population (case no. 101). Peak a, $G_0G_1$ of diploid cell population; peak b, $G_2$ + M of diploid cell population; peak c, $G_0G_1$ of aneuploid cell population; peak d, $G_2$ + M of aneuploid cell population

The detection of identical *p53* mutations in high-grade dysplasia and adjacent adenocarcinoma has been reported in two independent studies (Hamelin et al, 1994; Neshat et al, 1994). One study did not detect mutations in areas of low-grade dysplasia ($n$ = 3) and non-dysplastic Barrett's epithelium ($n$ = 6) adjacent to carcinoma (Hamelin et al, 1994). Similarly, in the present study, there was one case (no. 108) in which the *p53* mutation detected in high-grade dysplasia and tumour was not detected in adjacent low-grade dysplasia and non-dysplastic Barrett's epithelium. Therefore, to date, p53 protein expression and *p53* gene mutation have been detected more frequently in high-grade dysplasia than in low-grade dysplasia, suggesting that *p53* gene mutation may be associated with increasing severity of dysplasia.

Between 37.5% and 68% of gastric adenocarcinomas have been reported to demonstrate 17p allelic loss (Sano et al, 1991; Seruca et al, 1992; Ranzani et al, 1993). These values are lower than the value of 83% noted in the present series of adenocarcinomas arising in the gastric cardia. It is possible that site-specific differences (cardia vs antrum) may account for this higher frequency of allelic loss. Studies have reported *p53* mutations in 35–58% of gastric carcinomas (Renault et al, 1993; Uchino et al, 1993; Shiao

© Cancer Research Campaign 1998

et al, 1994; Hongyo et al, 1995), and p53 protein expression has been detected in 46–61% of tumours (Martin et al, 1992; Flejou et al, 1994; Hongyo et al, 1995). DNA aneuploidy has been reported in 40–50% of tumours (Yonemura et al, 1992; Brito et al, 1993). However, studies in which tumours were analysed with respect to anatomical site of origin have reported a number of site-specific differences. A significantly higher level of p53 expression was observed in gastric cardia tumours (56%, 20 of 36) compared with tumours arising in the antrum (27%, 8 of 30) (Flejou et al, 1994). Furthermore, a higher frequency of DNA aneuploidy was detected in adenocarcinomas arising in the gastric cardia (Johnson et al, 1993; Flejou et al, 1994). For example, one study reported aneuploidy in 76% (25 of 33) of adenocarcinomas arising in the gastric cardia, compared with 30% (8 of 27) of adenocarcinomas arising in the gastric antrum (Flejou et al, 1994). These molecular data are consistent with reports that proximal and distal gastric tumours display distinct clinical and epidemiological features. In general, carcinomas of the gastric cardia have a poorer prognosis than those of the gastric body, and their relative incidence appears to be increasing (Sidoni et al, 1989; Blot et al, 1991). Conversely, adenocarcinoma of the oesophagus and gastric cardia have been reported to demonstrate similar clinicopathological parameters. Both tumour types have demonstrated a parallel increase in incidence rates (Blot et al, 1991). These tumours have been consistently associated with a lower mean age, higher male–female ratio and greater frequency of hiatal hernia than distal gastric tumours (Kalish et al, 1984; Wang et al, 1986). The observation in this study of similar frequencies of *p53* gene abnormalities and DNA aneuploidy in these tumour types is consistent with the hypothesis that they share a common aetiology.

The occurrence of *p53* mutations early in the development of oesophageal and gastric adenocarcinoma (Shiao et al, 1994) suggests that the gene plays a central role in the control of normal cell division at these sites. The oesophagus and stomach are easily accessible and are likely to be exposed to many environmental insults. *p53*-dependent pathways may play a vital role in preserving molecular integrity by allowing damaged cells to undergo repair or apoptosis. Cells that have acquired a *p53* mutation may have a selective advantage, being able to proliferate under conditions of DNA damage that would be inhibitory in cells with wild-type *p53*. These actively proliferating cells may be the precursors of neoplastic clones.

The detection of frequent mutations in a defined sequence has been identified as a feature of mutational specificity (Thilly, 1990). It has been proposed that the comparison of *p53* mutation spectra in tumours of different origin may reveal similarities or differences concerning the endogenous and exogenous molecular processes contributing to tumour development (Greenblatt et al, 1994). The observation of similar *p53* mutation spectra in adenocarcinoma of the oesophagus and gastric cardia further suggests similarities in the aetiologies of these cancers. Both tumour groups demonstrated a predominance of base transitions at CpG dinucleotides and a low frequency of base transversions. G:C to A:T base transitions at CpG dinucleotides are thought to result from spontaneous deamination of 5′-methylcytosine, resulting in C to T replacements (Coulondre et al, 1978). Other studies have reported a predominance of base transitions at CpG dinucleotides in Barrett's-associated adenocarcinoma (Hamelin et al, 1994; Neshat et al, 1994). Furthermore, this spectrum of *p53* mutations is similar to that previously described for both colorectal cancer (Baker et al, 1990) and gastric cancer (Renault et al, 1993; Uchino et al, 1993). It has been reported that base transitions at CpG dinucleotides account for 62.5% (20 of 32) and 67% (8 of 12) of single-base substitutions in colorectal and gastric carcinoma respectively (Baker et al, 1990; Renault et al, 1993). Conversely, although a study by Hongyo et al (1995), in a high incidence area of gastric cancer in Italy, detected G:C to A:T transitions in 93% (26 of 28) of cases with single-base substitutions, only 18% (5 of 28) occurred at CpG dinucleotides. The different mutation spectrum in the study of Hongyo et al (1995) may reflect site-specific differences (cardia vs antrum) or regional exposure to particular environmental agents.

It is generally accepted that the majority of missense mutations generate a mutant protein with increased protein stability, thereby facilitating detection by immunohistochemical techniques (Cripps et al, 1994). Consistent with this, a highly significant association was observed between missense mutation of the *p53* gene and diffuse p53 protein expression in both oesophageal and gastric adenocarcinoma, with all 31 cases with missense mutations exhibiting diffuse protein expression. A previous study of oesophageal adenocarcinoma also reported a close correlation between missense mutation and p53 protein expression (100% agreement, 10 of 10) (Hamelin et al, 1994).

In the present study, there were six cases with diffuse p53 protein expression that were not found to contain a mutation in exons 5–8 of the *p53* gene. There are a number of possibilities that may account for the apparent discordance in these samples. Firstly, mutations may have been missed by the screening techniques used in the present study. Secondly, missense mutations may lie in regions of the gene not screened in the present study. A review of 50 studies that carried out sequencing of the entire coding region of the *p53* gene reported that 13% of mutations, up to 30% of which were missense mutations, were located outside exons 5–8 (Greenblatt et al, 1994). A third possibility is that mechanisms other than mutation resulted in the inactivation and stabilization of p53 protein. Binding to viral oncoproteins, e.g. the adenovirus E1B 55-kb protein, and cellular oncoproteins, e.g. mdm2, has been shown to result in p53 stabilization and in the inactivation of wild-type *p53* function (Yew and Berk, 1992; Wu et al, 1993). In these instances, p53 overexpression represents the functional, but not the structural, inactivation of the *p53* gene. In this respect, it was suggested that 'false positives' may not be misleading in the biological sense if the stabilization of p53 occurs via a mechanism that also abolishes its function (Wynford-Thomas, 1992).

None of 21 tumours with focal or negative immunostaining were found to have a missense mutation, suggesting that focal immunopositivity is not an indicator of mutational stabilization of the p53 protein. However, truncating *p53* mutations (non-sense mutations, insertions/deletions and splice site mutations) were detected in 14% (2 of 14) of oesophageal adenocarcinomas and in 27% (7 of 26) of gastric adenocarcinomas studied. All nine truncating mutations were immunonegative. These data indicate that a proportion of immunonegative tumours may contain a mutation that does not result in protein stabilization. It has been reported that truncating mutations may account for as much as 37.5% of mutations in oesophageal adenocarcinoma (Hamelin et al, 1994) and 27% of mutations in gastric adenocarcinoma (Renault et al, 1993; Uchino et al, 1993). In these cases, immunohistochemical assessment of p53 protein expression as a sole indicator of the presence of *p53* gene mutation would result in an underestimation of mutation frequency (false negatives). The observation of

© Cancer Research Campaign 1998

apparent discordance between molecular and immunohistochemical analysis of *p53* gene mutation indicates that such techniques should be regarded as complementary.

The acquisition of chromosomal rearrangements following the loss of p53 function has been observed in a number of in vitro systems (Livingstone et al, 1992; Yin et al, 1992). This suggests a potential association between *p53* gene abnormalities and the development of genetic instability (Livingstone et al, 1992). Evidence to support this hypothesis was provided by studies on colorectal carcinoma in which DNA aneuploidy was shown to be associated with 17p allelic loss (Offerhaus et al, 1992) and increased p53 protein expression (Carder et al, 1993). Furthermore, Carder et al (1993) reported that the mean DNA index in aneuploid tumours with stabilized p53 protein was significantly higher than that in those aneuploid cases without stabilized protein. In this study, statistical analysis demonstrated evidence of an association between the presence of 17p allelic loss and DNA aneuploidy (Fisher exact test, $P = 0.02$). However, the presence of *p53* gene mutation or p53 protein expression was not found to be significantly associated with the occurrence of DNA aneuploidy in oesophageal and gastric adenocarcinoma. Furthermore, the mean DNA index in aneuploid tumours with a *p53* mutation was not significantly higher than that in aneuploid cases without a *p53* gene mutation. It is uncertain to what extent the association between 17p allelic loss and abnormal DNA content reflects the involvement of p53 inactivation in the development or establishment of aneuploid cell populations.

For both the oesophageal and gastric tumour series studied, neither 17p allelic loss, *p53* gene mutation, p53 protein expression nor DNA aneuploidy exhibited an association with either tumour stage or histological subtype. This may reflect, in part, the predominantly advanced stage of tumours in the present series. Similarly, other studies on gastric adenocarcinoma have reported no association between DNA ploidy and depth of tumour invasion or lymph node metastasis (Yonemura et al, 1992; Brito et al, 1993). Studies have reported an equal prevalence of *p53* mutations in early- and late-stage gastric carcinoma (Uchino et al, 1993; Hongyo et al, 1995), and one study has reported the detection of the same *p53* mutation in both tumour and adjacent dysplastic epithelium in five cases of gastric adenocarcinoma (Shiao et al, 1994). In oesophageal adenocarcinoma, no association has been reported between p53 protein expression and tumour differentiation grade, tumour stage (Flejou et al, 1993; Krishnadath et al, 1995) or lymph node status (Flejou et al, 1993). Allelic loss on 17p, *p53* gene mutation and p53 protein expression have been documented in dysplastic Barrett's epithelium (Flejou et al, 1993; Blount et al, 1994; Hardwick et al, 1994) and in both early and advanced adenocarcinomas (Blount et al, 1991; Flejou et al, 1993; Hamelin et al, 1994). These data suggest that *p53* gene abnormalities may occur early in the development of oesophageal adenocarcinoma. The value of p53 as an intermediate biomarker in the identification of patients at increased risk of malignant transformation in Barrett's oesophagus awaits the outcome of prospective follow-up studies.

In conclusion, the present study demonstrated uniformly high levels of *p53* gene abnormalities and DNA aneuploidy in adenocarcinoma of the oesophagus and gastric cardia. Both oesophageal and gastric tumours exhibited similar *p53* mutation profiles, with both tumour types being associated with a high frequency of base transitions at CpG dinucleotides. The detection of similar molecular alterations in adenocarcinoma of the oesophagus and gastric cardia is consistent with the observation that these tumours also exhibit similar clinical and epidemiological features. There was a highly significant association between *p53* missense mutation and the presence of diffuse p53 protein expression in these tumours, supporting the rationale for using immunohistochemical methods as an indicator of missense mutation. However, truncating mutations, which may account for a significant proportion of *p53* mutations in oesophageal and gastric tumours, can not be detected by immunohistochemical techniques. Allelic loss on 17p, *p53* gene mutation and p53 protein expression were each detected in premalignant Barrett's epithelium. Future prospective studies should determine the clinical relevance of *p53* gene alterations as an independent marker of Barrett's patients at an increased risk of neoplastic transformation.

### ABBREVIATIONS

LOH, loss of heterozygosity; PCR, polymerase chain reaction; SSCP, single-strand conformational polymorphism

### ACKNOWLEDGEMENT

This work was supported by a grant from The Northern Ireland Chest, Heart and Stroke Association.

### REFERENCES

Baker SJ, Preisinger AC, Jessup JM, Paraskeva C, Markowitz S, Willson JKV, Hamilton S and Vogelstein B (1990) *p53* gene mutations occur in combination with 17p allelic deletions as late events in colorectal tumorigenesis. *Cancer Res* **50**: 7717–7722

Beahrs OH, Henson D, Hutter RVP and Myers MH (1988) *Manual for Staging Cancer, American Joint Committee on Cancer*. JB Lippincott: Philadelphia

Blot WJ, Devesa SS, Kneller RW and Fraumeni JF (1991) Rising incidence of adenocarcinoma of the esophagus and gastric cardia. *JAMA* **265**: 1287–1289

Blount PL, Ramel S, Raskind WH, Haggitt RC, Sanchez CA, Dean PJ, Rabinovitch PS and Reid BJ (1991) 17p allelic deletions and p53 protein overexpression in Barrett's adenocarcinoma. *Cancer Res* **51**: 5482–5486

Blount PL, Meltzer SJ, Yin J, Huang Y, Krasna MJ and Reid BJ (1993) Clonal ordering of 17p and 5q allelic losses in Barrett's dysplasia and adenocarcinoma. *Proc Natl Acad Sci USA* **90**: 3221–3225

Blount PL, Galipeau PC, Sanchez CA, Neshat K, Levine DS, Yin J, Suzuki H, Abraham JM, Meltzer SJ and Reid BJ (1994) 17p allelic losses in diploid cells of patients with Barrett's esophagus who develop aneuploidy. *Cancer Res* **54**: 2292–2295

Brito MJ, Filipe MI, Williams GT, Thompson H, Ormerod MG and Titley J (1993) DNA ploidy in early gastric carcinoma (T1): a flow cytometric study of 100 European cases. *Gut* **34**: 230–234

Carder P, Wyllie AH, Purdie C, Morris RG, White S, Piris J and Bird CC (1993) Stabilized p53 facilitates aneuploid clonal divergence in colorectal cancer. *Oncogene* **8**: 1397–1401

Coulondre C, Miller JH, Farabaugh PJ and Gilbert W (1978) Molecular basis of base substitution hotspots in *Escherichia coli*. *Nature* **274**: 775–780

Cripps KJ, Purdie CA, Carder PJ, White S, Komine K, Bird CC and Wyllie AH (1994) A study of stabilisation of p53 protein versus point mutation in colorectal carcinoma. *Oncogene* **9**: 2739–2743

Flejou J-F, Potet F, Muzeau F, Le Pelletier F, Fekete F and Henin D (1993) Overexpression of p53 protein in Barrett's syndrome with malignant transformation. *J Clin Pathol* **46**: 330–333

Flejou J-F, Muzeau F, Potet F, Lepelletier F, Fekete F and Henin D (1994) Overexpression of the p53 tumor suppressor gene product in esophageal and gastric carcinomas. *Path Res Pract* **190**: 1141–1148

Gleeson CM, Sloan, JM, McGuigan JA, Ritchie AJ and Russell SEH (1995) Base transitions at CpG dinucleotides in the *p53* gene are common in oesophageal adenocarcinoma. *Cancer Res* **55**: 3406–3411

Greenblatt MS, Bennett WP, Hollstein M and Harris CC (1994) Mutations in the *p53* tumor suppressor gene: clues to cancer etiology and molecular pathogenesis. *Cancer Res* **54**: 4855–4878

© Cancer Research Campaign 1998

Gyapay G, Morissette J, Vignal A, Dib C, Bernardi G, Lathrop M and Weissenbach J (1994) The 1993–1994 Genethon human genetic linkage map. *Nature Genet* **7**: 246–339

Haggitt RC, Reid BJ, Rabinovitch PS and Rubin CE (1988) Barrett's esophagus: correlation between mucin histochemistry, flow cytometry, and histologic diagnosis for predicting increased cancer risk. *Am J Pathol* **131**: 53–61

Hamelin R, Flejou J-F, Muzeau F, Potet F, Laurent-Puig P, Fekete F and Thomas G (1994) TP53 gene mutations and p53 protein immunoreactivity in malignant and premalignant Barrett's esophagus. *Gastroenterology* **107**: 1012–1018

Hardwick RH, Shepherd NA, Moorghen M, Newcomb PV and Alderson D (1994) Adenocarcinoma arising in Barrett's oesophagus: evidence for the participation of p53 dysfunction in the dysplasia/carcinoma sequence. *Gut* **35**: 764–768

Hedley DW, Friedlander ML, Taylor IW, Rugg CA and Musgrove EA (1983) Method for analysis of cellular DNA content of paraffin embedded pathological material using flow cytometry. *J Histochem Cytochem* **31**: 1333–1335

Hermanek P and Sobin LH (1987) *Classification of Malignant Tumors*. Springer: Berlin

Hongyo T, Buzard GS, Palli D, Weghorst CM, Amorosi A, Galli M, Caporaso NE, Fraumeni JF and Rice JM (1995) Mutations of the K-*ras* and *p53* genes in gastric adenocarcinomas from a high-incidence region around Florence, Italy. *Cancer Res* **55**: 2665–2672

Hsu IC, Metcalf RA, Sun T, Welsh JA, Wang NJ and Harris CC (1991) Mutational hotspot in the *p53* gene in human hepatocellular carcinomas. *Nature* **350**: 427–428

Huang Y, Boynton RF, Blount PL, Silverstein RJ, Yin J, Tong Y, McDaniel TK, Newkirk C, Resau JH, Sridhara R, Reid BJ and Meltzer SJ (1992) Loss of heterozygosity involves multiple tumor suppressor genes in human esophageal cancers. *Cancer Res* **52**: 6526–6530

Johnson H, Belluco C, Masood S, Abou-Azama AM, Kahn L and Wise L (1993) The value of flow cytometric analysis in patients with gastric cancer. *Arch Surg* **128**: 314–317

Kalish RJ, Clancy PE, Orringer MB and Appelman HD (1984) Clinical, epidemiologic, and morphologic comparison between adenocarcinomas arising in Barrett's esophageal mucosa and in the gastric cardia. *Gastroenterology* **86**: 461–467

Kastan MB, Onyekwere O, Sidransky D, Vogelstein B and Craig RW (1991) Participation of p53 protein in the cellular response to DNA damage. *Cancer Res* **51**: 6304–6311

Krishnadath KK, Tilanus HW, Van Blankenstein M, Bosman FT and Mulder AH (1995) Accumulation of p53 protein in normal, dysplastic, and neoplastic Barrett's oesophagus. *J Pathol* **175**: 175–180

Lauren P (1965) The two histological main types of gastric adenocarcinoma: diffuse and so-called intestinal-type adenocarcinoma. *Acta Pathol Microbiol Immunol Scand* **5**: 145–153

Livingstone LR, White A, Sprouse J, Livanos E, Jacks T and Tisty TD (1992) Altered cell cycle arrest and gene amplification potential accompany loss of wild-type *p53*. *Cell* **70**: 923–935

Martin HM, Filipe MI, Morris RW, Lane DP and Silvestre F (1992) p53 expression and prognosis in gastric carcinoma. *Int J Cancer* **50**: 859–862

Martinez J, Georgoff I, Martinez J and Levine AJ (1991) Cellular localization and cell cycle regulation by a temperature-sensitive p53 protein. *Genes Dev* **5**: 151–159

McBride OW, Merry D and Givol D (1986) The gene for human p53 cellular tumor antigen is located on chromosome 17 short arm (17p13). *Proc Natl Acad Sci USA* **83**: 130–134

Nakamura T, Nekarda H, Hoelscher AH, Bollschweiler E, Harbeck N, Becker K, Siewert JR and Harbeck H (1994) Prognostic value of DNA ploidy and c-erbB-2 oncoprotein overexpression in adenocarcinoma of Barrett's esophagus. *Cancer* **73**: 1785–1794

Neshat K, Sanchez CA, Galipeau PC, Blount PL, Levine DS, Joslyn G and Reid BJ (1994) *p53* mutations in Barrett's adenocarcinoma and high-grade dysplasia. *Gastroenterology* **106**: 1589–1595

Offerhaus GJA, De Feyter EP, Cornelisse CJ, Tersmette KWF, Floyd J, Kern SE, Vogelstein B and Hamilton SR (1992) The relationship of DNA aneuploidy to molecular genetic alterations in colorectal carcinoma. *Gastroenterology* **102**: 1612–1619

Oliphant AR, Wright EC, Swensen J, Gruis NA, Goldgar D and Skolnick MH (1990) Dinucleotide repeat polymorphism at the D17S513 locus. *Nucleic Acids Res* **19**: 4794

Powell J and McConkey CC (1990) Increasing incidence of adenocarcinoma of the gastric cardia and adjacent sites. *Br J Cancer* **62**: 440–443

Ranzani GN, Renault B, Pellegata NS, Fattorini P, Magni E, Bacci F and Amadori D (1993) Loss of heterozygosity and K-*ras* gene mutations in gastric cancer. *Hum Genet* **92**: 244–249

Reid BJ, Haggitt RC, Rubin CE and Rabinovitch PS (1987) Barrett's esophagus: correlation between flow cytometry and histology in detection of patients at risk for adenocarcinoma. *Gastroenterology* **93**: 1–11

Renault B, Van Den Broek M, Fodde R, Wijnen J, Pellegata NS, Amadori D, Khan PM and Ranzani GN (1993) Base transitions are the most frequent changes at *p53* in gastric cancer. *Cancer Res* **53**: 2614–2617

Sano T, Tsujino T, Yoshida K, Nakayama H, Haruma K, Ito H, Nakamura Y, Kajiyama G and Tahara E (1991) Frequent loss of heterozygosity on chromosomes 1q, 5q, and 17p in human gastric carcinomas. *Cancer Res* **51**: 2926–2931

Seruca R, David L, Holm R, Nesland JM, Fangan BM, Castedo S, Sobrinho-Simoes M and Borresen A-L (1992) *p53* mutations in gastric carcinomas. *Br J Cancer* **65**: 708–710

Shiao Y-H, Rugge M, Correa P, Lehmann HP and Scheer WD (1994) *p53* alterations in gastric precancerous lesions. *Am J Pathol* **144**: 511–517

Sidoni A, Lancia D, Pietropaoli N and Ferri I (1989) Changing patterns in gastric carcinoma. *Tumori* **75**: 605–608

Soussi T, De Fromentel CC and May P (1990) Structural aspects of the p53 protein in relation to gene evolution. *Oncogene* **5**: 945–952

Spechler SJ, Robbins AH, Bloomfield Rubins H, Vincent ME, Heeren T, Doos WG, Colton T and Schimmel EM (1984) Adenocarcinoma and Barrett's esophagus: an overrated risk? *Gastroenterology* **87**: 927–933

Thilly WG (1990) Mutational spectrometry in animal toxicity testing. *Annu Rev Pharmacol Toxicol* **30**: 369–385

Thompson JJ, Zinsser KR and Enterline HT (1983) Barrett's metaplasia and adenocarcinoma of the esophagus and gastroesophageal junction. *Hum Pathol* **14**: 42–61

Uchino S, Noguchi M, Ochiai A, Saito T, Kobayashi M and Hirohashi S (1993) *p53* mutation in gastric cancer: a genetic model for carcinogenesis is common to gastric and colorectal cancer. *Int J Cancer* **54**: 759–764

Wang HH, Antonioli DA and Goldman H (1986) Comparative features of esophageal and gastric adenocarcinomas: recent changes in type and frequency. *Hum Pathol* **17**: 482–487

Winters C, Spurling TJ, Chobanian SJ, Curtis DJ, Esposito RL, Hacker III JF, Johnson DA, Cruess DF, Cotelingam JD, Gurney MS and Cattau Jr EL (1987) Barrett's esophagus. A prevalent, occult complication of gastroesophageal reflux disease. *Gastroenterology* **92**: 118–124

Wu X, Bayle JH, Olson D and Levine AJ (1993) The p53-mdm-2 autoregulatory feedback loop. *Genes Dev* **7**: 1126–1132

Wynford-Thomas D (1992) p53 in tumour pathology: can we trust immunocytochemistry? *J Pathol* **166**: 329–330

Yew PR and Berk AJ (1992) Inhibition of p53 transactivation required for transformation by adenovirus early 1B protein. *Nature* **357**: 82–85

Yin Y, Tainsky MA, Bischoff FZ, Strong LC and Wahl GM (1992) Wild-type p53 restores cell cycle control and inhibits gene amplification in cells with mutant p53 alleles. *Cell* **70**: 937–948

Yonemura Y, Ohoyama S, Kimura H, Matumoto H, Ninomiya I, Kosaka T, Yamaguchi A, Miwa K and Miyazaki I (1992) Independent clinical and flow cytometric prognostic factors for the survival of patients with stage I gastric cancer. *Surg Today* **22**: 416–420

© Cancer Research Campaign 1998